# Hydrodynamics of the Certas™ programmable valve for the treatment of hydrocephalus

**DOI:** 10.1186/2045-8118-9-12

**Published:** 2012-06-29

**Authors:** Anders Eklund, Lars-Owe D Koskinen, Michael A Williams, Mark G Luciano, Stephen M Dombrowski, Jan Malm

**Affiliations:** 1 Department of Radiation Sciences, Umeå University, Umeå, S-901 85, Sweden; 2 Department of Clinical Neuroscience, Umeå University, Umeå, Sweden; 3 Department of Neurology, The Sandra and Malcolm Berman Brain & Spine Institute, Sinai Hospital, Baltimore, US; 4 Department of Neurosurgery, Cleveland Clinic, Cleveland, US

**Keywords:** Hydrocephalus, Normal pressure hydrocephalus, CSF, Cerebrospinal fluid, Shunt, Intracranial pressure, Outflow resistance, Conductance

## Abstract

**Background:**

The new Certas™ shunt for the treatment of hydrocephalus has seven standard pressure settings that according to the manufacturer range from 36 to 238 mmH_2_O, and an additional “Virtual Off” setting with an opening pressure >400 mmH_2_O. Information on actual pressure response and reliability of shunt performance is important in clinical application, especially the “Virtual Off” setting as a non-surgical replacement for shunt ligation. The objective of this study was to evaluate the *in-vitro* hydrodynamic performance of the Certas™ shunt.

**Methods:**

Six new Certas™ shunts with proximal and distal catheters were tested with an automated, computerized test system that raised the pressure from zero to a maximum pressure and back to zero at each valve setting. Opening pressure and flow resistance were determined.

**Results:**

For settings 1–7 the measured opening pressure range was 26 to 247 mmH_2_O, and the mean change in opening pressure for a one-step adjustment was between 33 and 38 mmH_2_O. For setting 8 (“Virtual Off”) the measured mean opening pressure was 494 ± 34 mmH_2_O (range 451 to 556 mmH_2_O). The mean outflow resistance was 7.0 mmHg/ml/min (outflow conductance 17.9 μl/s/kPa).

**Conclusions:**

The six shunts had similar characteristics and closely matched the manufacturer’s specifications for opening pressure at settings 1–7. The opening pressure for the “Virtual Off” setting was nearly 500 mmH_2_O, which is 100 mmH_2_O higher than the manufacturer’s specification of “>400” and should be functionally off for most patients with communicating hydrocephalus. Clinical studies are needed to evaluate if the CSF dynamic profile persists after implantation in patients.

## Introduction

Improvements in the modern shunt for drainage of cerebrospinal fluid (CSF) in the surgical treatment of hydrocephalus have aimed to include features in shunt design that reduce complications and improve clinical outcome. One goal has been to increase control over the amount of CSF drainage, such as adjustable shunt valves that permit postoperative adjustment of the shunt valve opening pressure. Several different brands of adjustable CSF shunts are available, characterized by opening pressures ranging from approximately 0 to 200 mm H_2_O, depending on the brand and the model. If a patient’s clinical response after shunt surgery is inadequate, lowering the shunt valve opening pressure may improve outcome while avoiding surgery to remove and replace the shunt valve. Alternately, in patients with over-drainage symptoms of headache or hearing change, or signs such as subacute or chronic subdural effusion or hemorrhage, treatment can be initiated by increasing the shunt valve opening pressure, thus avoiding surgery [1]. In several clinical settings, such as subacute or chronic subdural fluid collections, or efforts to achieve shunt independence, the clinician may prefer to stop CSF drainage. There is currently no valve system that provides this option, and even adjustable valves must be disabled by surgical ligation of the system because flow through the shunt is still possible at a valve opening pressure of 200 mmH_2_O.

The Codman Certas™ programmable valve was approved for clinical use in both Europe and the US in 2011. The Certas™ is an adjustable shunt with 7 pressure settings that range, according to the manufacturer, from 36 to 238 mm H_2_O. An interesting feature of the shunt is an eighth setting with a very high opening pressure (>400 mm H_2_O), that is described as “Virtual Off”. The aim of our study was to evaluate the hydrodynamic characteristics of the Certas™ programmable valve with an *in-vitro* bench test system.

## Methods

### The CSF shunts

Six new Certas™ shunts without a SiphonGuard™ were purchased from Codman (Wokingham, UK). The shunts were tested with the original 14 cm proximal and 120 cm distal catheters. The proximal (ventricular) catheter was shortened approximately two centimeter in order to remove the perforated part so that it could be attached to the test rig for perfusion.

### The test system

**Figure 1:**
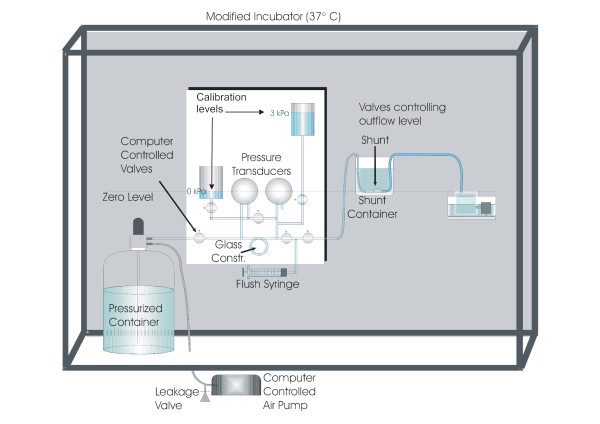
**The test system.** Proximal pressure of the shunt was controlled by air pressurizing the sealed bottle with the computer controlled air pump. Flow through the shunt was estimated from the pressure drop over the glass constriction using the calibrated resistance. Both the pressure drop over the constriction and the proximal shunt pressure were measured with differential pressure transducers from Druck (LPM8000). Outflow from the shunt was led into an overflow container with a constant water level at the zero pressure level. The set-up was placed in an incubator set at 37°C. Computer–controlled solenoid cocks were used to automatically perform the protocol, including pressure recalibrations.

The fully automated test system (Figure 1) used by the Umeå hydrocephalus group (http://www.hydrocephalus.se) has been described previously [2-4]. The system was updated with a new computer (Lifebook E780, Fujitsu, Tokyo, Japan) and a new data acquisition card (NI DAQCard-6036E, National Instruments, Austin TX,US). The software for the test system and analysis was developed in LabVIEW (National Instruments, Austin, TX, US). The computerized system collects data and regulates the pressure according to a pre-set pressure pattern. The inlet pressure to the shunt was regulated by air pressurizing a sealed 5 L bottle partially filled with water while measuring the proximal pressure of the CSF shunt with differential pressure transducers (LPM8000, Druck, Leicester, England). To prevent air bubbles, the de-ionized water in the test rig was first de-aerated by boiling for 10 min under vacuum. All tubing was visually inspected and any air bubbles were purged before the protocol start of each new shunt setting. Flow was calculated utilizing the principle of a differential pressure flow meter. Using an identical pressure transducer, the pressure drop across a glass constriction with a calibrated resistance was continuously measured. The flow rate is directly proportional to the pressure drop (Figure 1).

To simulate the effect of subcutaneous tissue pressure on the valve mechanism [5], the shunt was submerged in water at a depth of 100 mm. The distal catheter was led to an overflow container with a water level held constant at the zero pressure level to ensure a stable hydrostatic reference pressure. Because fluid viscosity and valve operating characteristics are temperature dependent, the test system was built into an incubator set at 37°C (Figure 1).

### Test protocol

Solenoid valves were computer controlled and the system performed all steps of the pre-programmed test protocol automatically, including a two-point pressure recalibration at zero and 305 mm H_2_O before testing at each valve opening pressure setting. For each valve setting, the inlet pressure was gradually increased from zero to a maximum pressure and then back to zero according to a triangular shaped waveform with a cycle period of 60 min [4]. The triangular wave was repeated 6 times at each setting for each valve for a total of 288 cycles. If air bubbles were detected during a cycle, the cycle was omitted (304 cycles with 16 omissions were necessary to achieve 288 cycles). Every shunt was tested at all eight opening pressure settings.

**Figure 2:**
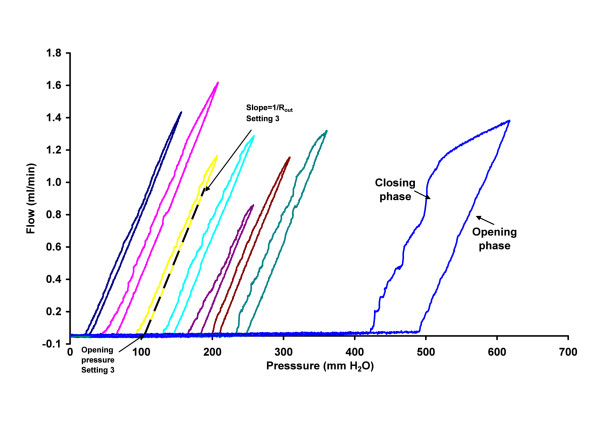
**Typical pressure-flow curves for settings 1 – 8 in order from left to right.** For each shunt setting, the valve opening pressure is determined during the rising phase of the inlet pressure. When the pressure enters the decreasing phase of the inlet pressure, the flow reduces, but with some hysteresis so that the closing pressure of the valve is lower than the opening pressure. The hysteresis was more pronounced for setting 8. The calculation for estimation of R_out_ and opening pressure is exemplified by the dotted line fitted against the opening phase of data from setting 3. The slope of the curve gives 1/R_out_ and the intersection of the line with the pressure axis is the opening pressure.

Typical flow versus pressure curves can be seen in Figure 2. The figure also shows hysteresis of the pressure-flow curve at each setting. A higher pressure is required to open the valve and sustain flow during the rising phase of the inlet pressure, but during the decreasing phase of the inlet pressure, the flow is sustained at a lower pressure so that the closing pressure of the valve is slightly lower than the opening pressure. To ensure that the flow rate reached at least 0.9 ml/min, used in the calculation for flow resistance and opening pressure (see Statistics and Figure 2), independent of shunt setting, while at the same time avoiding very high flow rates at low pressure settings, a maximum pressure was manually determined for each setting before starting the automatic protocol. In the figure it can be seen how different settings required different maximum pressures.

### Statistics

Opening conductance (1/R_out_) was determined as the slope of a linear regression between 0.45 and 0.9 ml/min (Figure 2). The shunt valve opening pressure was considered to be the pressure value at the intersection of the regression line with the x-axis (i.e., zero flow). Results at each shunt setting are presented as the mean value of 6 cycles per shunt for all 6 shunts.

To test for differences between groups analysis of variance (ANOVA) with Bonferroni Post Hoc test were used. *p* < 0.05 was considered statistically significant.

## Results

**Figure 3:**
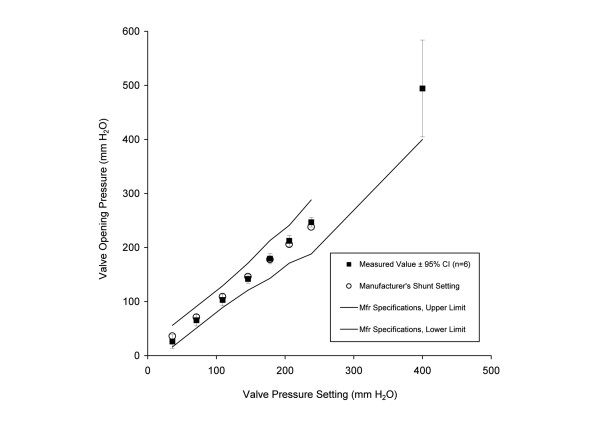
**Measured value of shunt opening pressure for the 8 settings (solid black squares) and the manufacturer’s specifications for each shunt setting (open black circles) plotted against the manufacturer’s specification for opening pressure.** Error bars show the 95% confidence level based on the six shunts. Tolerance limits for each shunt setting per manufacturer’s specifications are shown as solid lines for comparison. For the highest setting, only the lower limit of opening pressure > 400 mm H_2_O is specified by the manufacturer; thus, no upper tolerance limit is shown.

The shunt valve opening pressures were significantly different for settings 1 – 7 (*p* < 0.001, n = 42, ANOVA) and post hoc analysis showed that all settings were significantly different from each other. The mean change in opening pressure for a one-step adjustment was between 33 and 38 mm H_2_O for settings 1 to 7 (Figure 3). The measured opening pressure at the lowest setting (setting 1, 36 mm H_2_O) was 26 mm H_2_O and the measured opening pressure at the highest (setting 7, 238 mm H_2_O) was 247 mm H_2_O. For setting 8 (“Virtual Off”, >400 mm H_2_O), the measured mean opening pressure for the six shunts was 494 ± 34 mm H_2_O (range 451 to 556 mm H_2_O) and significantly higher than setting 7 (n = 12, *p* < 0.001). Because the 60 min cycle for the triangular pressure waveform produces the shunt’s *undisturbed* pressure-flow characteristics, the opening pressure determined by this test for each setting should be interpreted as a *maximum* operating pressure for that setting.

**Figure 4:**
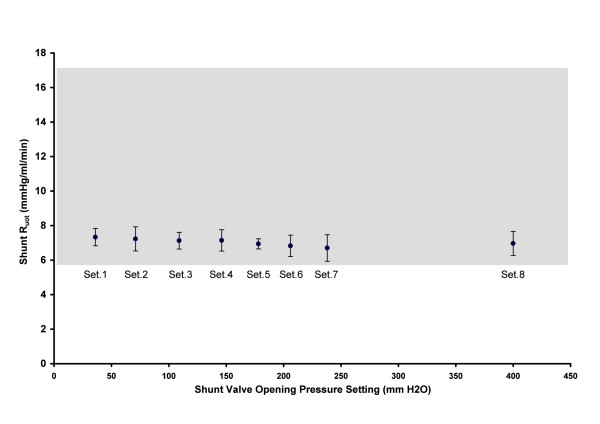
**Shunt outflow resistance for six shunts at each of the 8 shunt settings (shown in the labels).** Error bars show the 95% confidence limits. The shaded area shows the reference range (10^th^ to 90^th^ percentile) of Rout for healthy elderly [6].

The shunt outflow resistance (resistance to flow through the system) was dependent on the shunt setting (*p* < 0.01, n = 48, Figure 4). Post hoc analysis showed that settings 1 and 2 differed from settings 6 and 7, although the differences were small, varying from 6.7 mmHg/ml/min for setting 1 to 7.3 mmHg/ml/min for setting 7. The mean shunt outflow resistance was 7.0 ± 0.2 mmHg/ml/min (mean ± SD), corresponding to an outflow conductance of 17.9 ± 0.5 μl/s/kPa .

## Discussion

This *in vitro* test of the Certas™ valve has demonstrated a reliable, consistent step-wise regulation of opening pressure with an acceptable outflow resistance for all shunt valve settings. The six tested shunts had similar characteristics and were well within the manufacturer’s specifications for opening pressure at settings 1–7, with no overlap between settings (Figure 3). For settings 1-7, the opening pressure ranged from 26 to 247 mm H_2_O. The increment between settings was 33–38 mm H_2_O (≈2.7 mm Hg), which is larger than the 10 mm H_2_O (0.7 mm Hg) increment for the Codman Hakim shunt [4] and more similar to increments for the Medtronic Strata shunt [2]. For setting 8, “Virtual Off”, we determined the opening pressure was nearly 500 mm H_2_O, which is 100 mm H_2_O higher than the minimum of 400 mm H_2_O specified by the manufacturer. With the shunt implanted in patients, the cardiac related pulsations of intracranial pressure (ICP), in combination with the hysteresis characteristics of the valve (Figure 2) will probably result in the shunt operating within the boundaries of the hysteresis curve at each setting. Thus, the opening pressure of the shunt in a patient can be expected to be 10-25 mm H_2_O lower than the opening pressure determined by the test system.

In principle, the shunt’s main function is to create a CSF outflow pathway parallel to the patient’s CSF pathways, which are impaired and have increased CSF outflow resistance that plays a role in the pathophysiology of hydrocephalus. To anticipate an individual patient’s CSF dynamics after shunt surgery, it is essential to know the shunt operating characteristics and the patient’s preoperative CSF dynamics, which can be determined with an infusion test [3,7]. R_out_ >18 mmHg/ml/min is considered indicative of a response to shunting in patients with idiopathic normal pressure hydrocephalus (iNPH) [8]. The most important parameters for the shunt are the outflow resistance when the shunt is open, and the opening pressure of the valve at each setting.

The shunt outflow resistance describes the relationship between pressure and flow when the valve is open. The mean R_out_ is 7.0 mmHg/ml/min for the Certas™, and the variation between settings (6.7 to 7.3 mmHg/ml/min), while statistically significant, is small enough from a clinical perspective to be regarded as independent of the valve setting. In the test system, the mean R_out_ comprises the sum of R_out_ from the proximal catheter, the valve mechanism, and the distal catheter. From specifications provided in the shunt package insert, we can calculate that (1) the shunt valve R_out_ should be approximately 1.3 mmHg/ml/min, (2) the distal catheter R_out_ should be approximately 5.0 mmHg/ml/min, and if the proximal catheter resistance was approximately 0.7 mmHg/ml/min, the combined value is equal to the 7.0 mmHg/ml/min measured in this study. This is slightly higher than most shunts on the market [2-4].

The shunt outflow resistance of the Certas™ programmable valve (7.0 mmHg/ml/min) is lower than the physiological mean outflow resistance reported for patients with NPH (17.6 mmHg/ml/min) [9] and just below the median R_out_ (8.6 mmHg/ml/min) reported for healthy elderly [6]. Therefore, in a patient with a shunt, the shunt will usually be the path of least resistance for CSF outflow, and it will dominate the CSF pathways, creating a low resistance CSF dynamic system whenever the valve is open. As a result, measurement of CSF outflow resistance can be used to determine if a shunt is functioning or obstructed. The R_out_ of a patient with an obstructed shunt is high, and usually similar to the patient’s pre-shunt R_out_[10,11]. In Europe, when patients are evaluated for suspected hydrocephalus, infusion testing is often used to characterize the CSF dynamic system to determine whether R_out_ is abnormal [12] and shunt malfunction is present [10,13]. The expected R_out_ in a patient with a functioning Certas shunt should be in the range of 4.0 to 6.5 mmHg/ml/min (conductance 20 to 31 μl/s/kPa).

The opening pressure is the differential pressure across the valve mechanism needed for the valve mechanism to open. For example, with a ventriculo-peritoneal shunt configuration, for the valve to open when the patient is horizontal, the difference between the CSF pressure and the downstream pressure, which is the intra-abdominal pressure, must be greater than the valve opening pressure.

Recent reports support the use of a high valve setting on adjustable shunts as a noninvasive method to treat subacute or chronic subdural fluid collections or hematomas that have resulted from over drainage [14]. The opening pressure of settings 6 and 7 of the Certas™ valve are both higher than the opening pressure of the highest setting of the Codman Hakim [4] and Strata shunts [2]. We confirmed that the Certas™ valve setting 8, “Virtual Off”, has an opening pressure range of 451 to 556 mm H_2_O, which is significantly higher than the highest shunt setting of other adjustable valves, and can probably for most patients be regarded as functionally closed. Other potential uses for such high shunt settings include gradually raising the shunt setting in an attempt to make a patient shunt independent, or using the system as a “back up” after endoscopic third ventriculostomy (ETV), where the shunt would open at a very high CSF pressure that would occur if the ETV were to fail. The availability of higher opening pressures may also obviate the need for subsequent surgical implantation of an additional resistance device (anti-siphon device) in some patients. The proSA® shunt from Miethke has a comparable solution to the high pressure setting with an adjustable anti-gravitational device which can be adjusted to a counter pressure up to 400 mm H_2_O. However, in contrast to Certas™, the proSA® is only active in the upright position and in the supine position the opening pressure of that device is zero. Shunt system flow is then dependent on the opening pressure of the standard differential valve placed in series with the proSA®. Although not evaluated in this study, the Certas™ shunt system is available with the SiphonGuard™ anti-siphon device. In a previous study [4] we found that in the supine position neither the opening pressure nor the resistance was changed in the Codman Hakim valve system by adding the SiphonGuard™, nor were they affected by positioning the SiphonGuard™ either 10 cm above or 20 cm below the ventricular catheter tip. We expect that the same will hold for the Certas™ because the basic differential-pressure shunt design is the same.

An important question is whether the “Virtual Off” setting is likely to be functionally off in a patient. Portnoy *et al.* suggested that the perfusion pressure (PP) through the shunt is equal to: ICP + hydrostatic pressure – intra abdominal pressure – shunt opening pressure [15]. The PP must be greater than zero for the valve to open and CSF to flow. In the supine position the hydrostatic pressure is zero. Abdominal pressure is normally in the range of 70 to 190 mm H_2_O, and is dependent on obesity [16]. ICP in healthy elderly is 100 to 196 mm H_2_O [6] and in iNPH patients it is lower than 240 mm H_2_O [17]. In overnight monitoring, ICP is shown to be slightly higher during sleep, but periods of ICP above 205 mm H_2_O are rare in communicating hydrocephalus [18]. Plateau waves with large ICP increases could cause shunt flow, but they are not a typical feature in NPH patients [20]. Thus, in the supine position, using the limits of normal values for each variable and an opening pressure of 400, we can calculate that PP = (240 + 0 – 70 - 400) = −230 and no CSF will flow through the Certas™ shunt.

In the sitting position ICP is approximately zero [18-20]. Abdominal pressure on average increases with 120 mm H_2_O in the 45° sitting position [16] and in NPH patients the abdominal pressure in the sitting position is between 150 mm H_2_O [20] and 240 mm H_2_O [19]. The hydrostatic pressure will of course depend on the subject, but can be assumed around 500 to 600 mm H_2_O. The worst case scenario for upright PP = (0 + 600 – 150 - 400) = 50, which means that the Certas™ shunt valve could open. This shows that this limit is tight and that there is still risk for shunt flow. However, extrapolating the data from Miyake *et al.*[19], who measured ICP and abdominal pressure with different shunt settings up 200 mm H_2_O, to a shunt opening pressure of 400 mm H_2_O, indicates that the flow would be zero in all but one of their patients. Considering that the Certas™ shunt in this study had a mean shunt opening pressure above the limit of 400 mm H_2_O for all shunts and that in individual subjects the body probably “self compensates” for a larger hydrostatic pressure gradient with a larger increase in abdominal pressure for a taller person, we believe that the “Virtual Off” setting in patients with iNPH should act essentially as an off setting for most patients. However, we emphasize that this needs to be verified in the clinical setting.

The “Virtual Off” setting has applicability for research protocols. This setting could be used to non-invasively turn on or off the shunt in a blinded protocol, which previously has required either surgical clipping of the shunt catheter at the time of implantation [21] or implantation of a “dummy” shunt with an internal occlusion [22]. Reversal of the placebo condition in these studies required an additional surgical procedure, which is a significant risk from the perspective of research ethics, and may be a barrier to recruitment of research subjects. A blinded study design with randomization either to “Virtual Off” or usual functional opening pressure settings could determine the true clinical effect of shunt surgery, as well as determination of cerebral blood flow and metabolic responses induced by the changed CSF dynamics from the active shunting. The Certas™ is designed to prevent the shunt setting from changing in strong external magnetic fields, such as those associated with MRI [23], which would prevent inadvertent change of the shunt setting during the study protocol should the patient require an “off protocol” MRI for clinical purposes. Another important feature for the clinician is that, similar to the Medtronic Strata shunt the setting of the Certas™ shunt can be checked with an indicator tool, thereby avoiding unnecessary x-rays.

It should be noted that flow in the “Virtual Off” position has been considered here largely in normal and iNPH patients. In children and adults with typical “high” pressure hydrocephalus or with pseudotumour cerebri, flow may be present even at this setting. While this is likely a positive safety feature for patients with potentially high pressures and unlikely to impede its use for treatment of low pressure problems like subdurals, it should be realized that under these circumstances shunt removal or ligation differs from the “Virtual Off” setting.

## Conclusions

In conclusion we confirmed that the opening pressures and outflow resistance of the Certas™ adjustable valve closely matched the manufacturer’s specifications; and that pressure measured at the “Virtual Off” setting exceeded 400 mm H_2_O for all shunts. The “Virtual Off” setting may be useful in clinical situations where a reversible and non-invasively “closed” shunt may be desired. The “Virtual Off” feature may reduce the need for surgery in the treatment of subdural hygromas and hematomas, failed third ventriculostomies and shunt weaning.

## Competing interests

The Certas™ shunts were purchased from Codman Inc at a discounted price. The company did not claim any service in return. A Eklund and L-O D Koskinen have received honorarium for lecturing from DePuy Ltd (Codman Inc). M Luciano has (2005) received a clinical research grant from Codman, Inc. M Williams receives grant support from NeuroDx Development for work related to SBIR R43NS067770-01A1.

## Authors’ contributions

AE, JM and LODK participated in conception and design of the study and in collection of data. All authors contributed in analysis and interpretation of data, manuscript preparation, reviewed the final version of the manuscript and approved it for submission.
